# Institutional analysis of health promotion for older people in Europe - concept and research tool

**DOI:** 10.1186/s12913-016-1516-1

**Published:** 2016-09-05

**Authors:** Stojgniew J. Sitko, Iwona Kowalska-Bobko, Anna Mokrzycka, Michał Zabdyr-Jamróz, Alicja Domagała, Nicola Magnavita, Andrea Poscia, Maciej Rogala, Anna Szetela, Stanisława Golinowska

**Affiliations:** 1 Department of Health Policy and Management Institute of Public Health, Faculty of Health Sciences, Collegium Medicum, Jagiellonian University, Kraków, Poland; 2 Department of Public Health, Università Cattolica del Sacro Cuore, Rome, Italy

**Keywords:** Health promotion, Older population, Healthy aging, Health policy, Public social institutions, Sectorial approach, Health promotion programmes and policies, Institutional approach

## Abstract

**Background:**

European societies are ageing rapidly and thus health promotion for older people (HP4OP) is becoming an increasingly relevant issue. Crucial here is not only the clinical aspect of health promotion but also its organisational and institutional dimension. The latter has been relatively neglected in research on HP4OP. This issue is addressed in this study, constituting a part of the EU project ProHealth65+, engaging ten member countries. This paper is based on two intertwining research goals: (1) exploring which institutions/organisations are performing HP4OP activities in selected European countries (including sectors involved, performed roles of these institutions, organisation of those activities); (2) developing an institutional approach to HP4OP. Thus, the paper provides a description of the analytical tools for further research in this area.

**Methods:**

The mentioned aims were addressed through the mutual use of two complementary methods: (a) a literature review of scientific and grey literature; and (b) questionnaire survey with selected expert respondents from 10 European countries. The expert respondents, selected by the project’s collaborating partners, were asked to fill in a custom designed questionnaire concerning HP4OP institutional aspects.

**Results:**

The literature review provided an overview of the organisational arrangements in different HP4OP initiatives. It also enabled the development of functional institutional definitions of health promotion, health promotion activities and interventions, as well as an institutional definition adequate to the health promotion context. The distinctions between sectors were also clarified. The elaborated questionnaires provided in-depth information on countries specifically indicating the key sectors involved in HP4OP in those selected countries. These are: health care, regional/local authorities, NGO’s/voluntary institutions. The questionnaire and literature review both resulted in the indication of a significant level of cross-sectorial cooperation in HP4OP.

**Conclusions:**

The inclusion of the institutional analysis within the study of HP4OP provides a valuable opportunity to analyse, in a systematic way, good practices in this respect, also in terms of institutional arrangements. A failure to address this aspect in policymaking might potentially cause organisational failure even in evidence-based programmes. This paper frames the perception of this problem.

## Background

European societies are undergoing rapid demographic changes. Thus, for contemporary societies, health promotion for older people (HP4OP) is becoming a highly relevant issue. The literature confirms that the older population has rarely been a target of significant dedicated health promotion programmes. The study presented here mainly focuses on the questions of who, how and in which way provides HP4OP activities in European countries. Currently, the lack of a systematic, comparative cross-country institutional analysis of that topic is evident. Moreover, a dedicated methodological approach to the topic does not seem to be being performed [1]. This paper addresses the research gap outlined above.

The basic assumption here is that the institutional environment and organisation constitute crucial components of the implementation of any policy in this field, especially in the context of capacity building for a greater sustainability of health promotion. The paper is based on the two intertwining research goals. Firstly, it explores the issue of which institutions/organisations perform HP4OP activities in selected European countries by detailing which sectors are involved, what kind of roles the institutions perform, and how they organise such activities. Secondly, it develops an institutional approach to HP4OP and provides the analytical tools for further research in the area by developing dedicated definitions and classifications. For the purpose of this study, i.e. an institutional approach to HP4OP, we define the key terms below. The definitions are a result of the research performed and are explained thoroughly in the results section.

*Health promotion* is the process of improving population health status by enabling the individuals to leave healthy within a community and through government interventions, i.e. to increase the control over their health and its determinants. It encompasses prevention, education, and advocacy. Health promotion should respect human autonomy, be sustainable, evidence-based and adjusted to the specificity of the target group and local context. It should also be performed as a concerted action by various entities belonging to all sectors and on all levels of governance in order to effectively engage the available resource.

Health promotion *activities* (the processes of health promotion as defined above) can take various forms. The most general is an *intervention* in the current state of affairs aimed at a behavioural, social, environmental or policy change. A health promotion *programme* is a set of organised activities strictly designed with a health promotion objective in mind, which are limited in terms of their scope, organisation, engaged actors and duration.

Finally, we define an institution as “an interlocking double-structure of persons-as-role-holders or office-bearers and the like, and of social practices involving both expressive and practical aims and outcomes”[2].

The above definitions are applied in our analysis to compare the provision of HP4OP activities in selected European countries. The analysis constitutes a part of the research carried out in the EU project ProHealth65+, which engages ten EU member states. These countries are also included in our analysis.

## Methods

The objectives described in this paper were addressed with the mutual use of two complementary methods: (a) a literature review of the scientific and grey literature; and (b) questionnaire surveys with selected expert respondents from the ten project countries. The literature review took into account the theoretical and interdisciplinary nature of the study. The expert respondents, selected by the project’s collaborating partners, filled in a custom designed questionnaire on the institutional aspects of HP4OP in their countries. Below, we first present a general overview of the study approach, and details about the two methods applied.

The countries included in the study allow obtaining a broader overview of the problem as they represent different parts of Europe, varying in welfare and healthcare models. The countries include: Bulgaria, the Czech Republic, Germany, Greece, Hungary, Italy, Lithuania, the Netherlands, Poland and Portugal. The project countries represent different institutional regimes of the welfare state and can be grouped in three main models corresponding to particular regions: (a) continental European countries, (b) southern European countries and (c) CEE countries. The health status of the older populations varies from country to country. The differences in the health status of older populations are also of a great significance. The study was based on collecting information about HP4OP activities and was carried out in 2015 in the analysed countries. This served as a based to systemise the knowledge on HP4OP practices in these EU countries and to indicate sources for examples of good practices.

Based on the analysis of the country experts’ opinions and the information given, preceded and supported by an extended literature review, the following sectors were chosen for comprehensive analysis: health, social assistance, government (central), regional/local authorities, voluntary/NGO, education, sport, and media. These sectors were indicated as dominating the sphere of health promotion activities in general. This paper concentrates on a description of the complex institutional picture of health promotion activities focused on older people in the analysed European countries, taking into account the above mentioned sectorial pre-conditions.

### Literature review

The initial knowledge concerning institutions acting in HP4OP in selected countries was acquired as a result of a literature review. The main sources of information were scientific papers and grey literature as well as other materials: government websites, strategic documents, programmes and projects, guidelines and other publicly sources that were accessible. Notable sources were reports developed by other institutions [3–21]. Previous literature reviews were profiled for theoretical background as well as for specificity to selected countries.

**Fig. 1:**
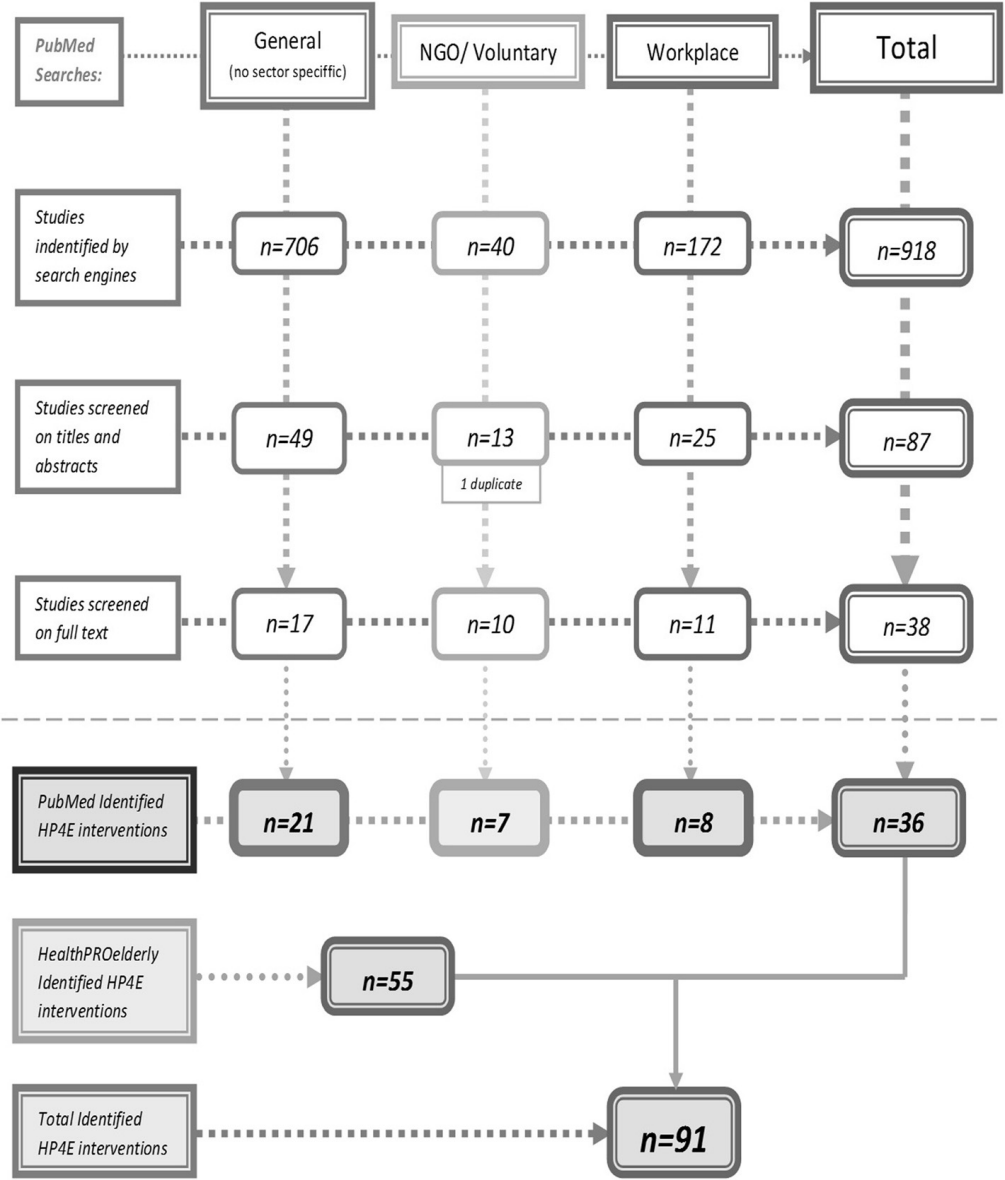
Literature Review Process

A literature review on countries’ institutional specificity was performed for English-language papers on HP4OP published between 2000 and 2015 in the project countries. The database selection was limited to PubMed and healthPROelderly database due to the scope of the research. This selection, provided a much needed comparison between scientific and grey literature sources. Search terms were selected in order to retrieve as many relevant sources as possible. Three sets of search terms were distinguished due to sector’s specificity: see Fig. 1. Two independent researchers performed the source selection in two stages: by abstract and full text screening. For detailed information on the literature search flow see Fig. 1. Publications not explicitly mentioning health promotion, focused on treating diseases, not explicitly addressing the target group of the older people, or with a purely medical-care focus were excluded. Publications concerning the screening or clinical evaluation of projects, as well as studies on ageing populations – observational studies such as EPOSA [20] – were included in order to identify institutions that study, monitor, evaluate and produce evidence-based knowledge for HP4OP [21]. Programmes aimed at the general population, oriented towards diseases, for which old age is a risk factor [22], were also included.

It was assumed, however, that some relevant institutional information was present in sources that could not be identified by the search engines mentioned above. Data provided by reviews on countries’ institutional specificity mostly required further specifications. For this purpose, a follow-up narrative review of other available sources (including the grey literature) was performed for the proper identification of the institutions mentioned (their character, status, sector classification, etc.). The goal of the reviews was limited to the identification of institutions involved in health promotion initiatives and their roles; thus a quality check of the publications reviewed was not necessary. A supplementary review was also performed using:

- journal databases: Health Policy Journal, Public Health, Journal of Public Health, and Health Systems in Transition [23–29];
- web search engines (e.g. Google) for websites, grey literature, and other non-indexed sources;
- websites of institutions dealing with issues related to the topic (promoters, providers, stakeholders, public agencies, NGOs, etc.);
- websites of responsible authorities (by selected states – after initial review) and the HealthPROelderly Project database [13].

Detailed information on the number of relevant publications identified during the above literature search phases is shown in Fig. 1. The publications were analysed following the method of content analysis. Specifically, the content of the publications was reviewed to extract information related to the research goals specified at the outset of the paper.

### Questionnaire survey

The first methodological question in the survey concerned the identification of institutions and organisations involved in HP4OP, in order to select those who are the most active in this field. Another important question concerned the research structure, determined by the involvement of many different project countries with a diversity of health promotion providers which had to be taken into account.

The areas to be analysed were indicated as follows: the programmes and interventions undertaken by different institutions within the given health care and public health system as well as other subjects situated outside of these (usually public/state) areas of responsibility. Attention was also paid to preventive measures, which led to the inclusion of occupational health promotion issues and issues related to the situation of older workers (occupational health usually relates to persons younger than 65). Another issue that appeared important for completing the picture was the media involvement in health promotion (TV, radio, press, and new media – Internet), which might play a significant role, especially in health marketing. The role played by education and sport institutions also needed to be taken into account.

The institutional approach was further enhanced, in light of competencies concerning health promotion activities, by the basic differentiation of research into three main stages: (A) health policy concept, creation, standards and plans (using necessary instruments, procedures and supporting incentives of different kinds); (B) policy/strategy introduction into the system (formal – in the form of adopting legislation or executive regulations, and declaratory – in the form of different policy documents, strategic plans, guidelines, standards and programmes); (C) implementation of policy, practical application/introduction and monitoring, control and surveillance, and the final process of assessment and evaluation. The analysis presented here was performed in the following two major steps: (I) identification of sectors most engaged in HP4OP in a group of EU countries, and (II) acknowledgment of country specific sectors and institutions supported by the survey.

A dedicated survey instrument – a questionnaire with a range of specific questions – was prepared in order to supplement and confirm the information for the country-specific perspective regarding the engagement of selected sectors in HP4OP activities obtained from the literature review. Bearing in mind that the information and data to be collected, were supposed to relate to a broader scope of health promotion, two aspects were addressed to the respondents: health promotion in general and HP4OP specifically. This meant that most of the questions had two sections for answers: relating to health promotion in general, and HP4OP specifically. The expert respondents, selected by the project’s collaborating partners, were also asked to fill in the questionnaire concerning HP4OP institutional aspects.

The pilot version of the questionnaire was consulted and pre-tested with project partners in Germany, the Netherlands and Italy. After receiving some minor feedback, its fine-tuned, final version was distributed among the expert respondents indicated by the project partners in the ten European countries. The questionnaire was sent by mail. The questionnaire itself and all communication with experts were conducted in English. All countries’ responses have been received and reviewed. The applied version of the Questionnaire may be found on the project website: http://pro-health65plus.eu. No language problems have been noticed. The whole process took about four months.

The final version of the questionnaire was composed of ten main questions (see Table 1) with empty fields for answers, mostly divided into 2 sections (health promotion and HP4OP), as mentioned above. In order to ensure clarity for respondents and to provide better structure concerning the responses, the questions were located within a table in a word-processor file. The list of questions, as they were posed in the questionnaire, is presented in Table 1.

**Table 1:** The English wording of the questions included in the questionnaire

Questions
*1) Indicate the 2–3 most important sectors in your country where there are institutions/organisations providing health promotion functions generally, as well as Health Promotion specifically addressed towards older people.*
*2) Identify the 2–3 most important, particular institutions/organisations affecting health promotion functions generally and addressed at older people in a given country in sectors indicated previously (in section (1)).*
*3) Are there any regulations formally obligating health promotion activity in your country (e.g., Public Health Act, Health Promotion Act)? If yes, indicate the names of those acts, year(s) when they were passed and links. If there are such regulations that only apply to particular sector(s), then please indicate below the name of the sector and the relevant names of acts, and year(s) when they were passed and links.*
*4) Is there in force, a country-wide, general, official, long-term National health programme (Strategy, Plan…)? If so, indicate its name, time scope and year when it was passed; if NOT – please indicate other relevant sectorial programmes/strategies/plans.*
*5) If such an official act or strategy/plan exists – does it encompass issues of Health Promotion addressed towards the older people? (Refers to HP4OP column only).*
*6) If there is/are regulation(s) as above (in section (4)) – do they enumerate/indicate any specific groups of people towards whom Health Promotion should be addressed?*
*7) If such a programme(s) exist(s) – what is its/their practical transmission (real causative influence) on the functioning of the organisations carrying out (or obliged to carry out) Health Promotion activities addressed towards older people (refers to HP4OP column only).*
*8) How are health promotion activities funded in your country? Who is the main public funder of those Health Promotion activities? Are there any non-public financial resources spent on them?*
*9) Are there evidence-based knowledge approaches used in the planning and shaping of the main Health Promotion programmes? Which kind of expertise is used? Who provides it?*
*10) Which are the most relevant national documents (reports, surveys, analysis, papers, etc.) which may be helpful for supplementing/enlarging the above information concerning the country?*

The first part of the survey (questions 1 and 2 in Table 1) aimed at identifying the sectors and particular organisations most active in health promotion in general and HP4OP in particular. The respondents had eight possibilities listed there: seven sectors – health, social, government (central), regional/local authorities, voluntary/NGO, education & sport and media, as well as an empty space to indicate other sector(s) if so required.

Questions 3 through 7 (see Table 1) were focused on the existence of formal regulations and official documents such as legislation, strategies and national plans – relating to both health promotion in general and HP4OP specifically in a given country. This was done in order to get information concerning the presumed diversity of the formal approach to this issue. The last question in this section was only related to HP4OP and concerned respondents’ opinions about the real causative influence of the organisations oriented towards older people.

Again, as a consequence of the supposed variety in different countries, question number 8 referred to the main sources of public and private financing of health promotion and HP4OP activities. Thus, respondents were asked whether there was an evidence-based approach to planning and shaping health promotion programmes – and if yes, how it was provided and by whom. Finally, there was a request to indicate the sources of data for the most relevant national documents. This was expected to help in completing or increasing the above information concerning a particular country. Respondents were also provided with a closing section for any additional comments or supplementary information.

The main results obtained from this survey, as well as the final conclusions – which give equal weight to the analysis of the literature review – were analysed in view of the research goals of the paper.

## Results

### Literature review results: institutional approach to HP4OP

The literature review provides an analytical insight into the organisational aspect of HP4OP. First of all, the great differences between countries should be indicated here, with regard to the institutional dimension, the structure and nature of the institutions involved, the scope of competencies, the scale of activities, the size of the institutions themselves, and, of course, the financial resources available (especially in respect to health promotion).

The institutional definition of health promotion is commonly based on two compounds: the first one focuses on the different activities undertaken in the field of health promotion (the functional approach – see Table 2), whereas the second concentrates on the different kinds of institutions engaged in the problem (the sector typology approach – see Table 3). Regarding the main activities in question, further division into two categories may be suggested accordingly to the general and specific addressees, i.e., the population as a whole and the older population. In relation to the second category and based on the WHO documents, there are different kinds of health promotion activities specifically dedicated to the older generation, such as walking for health, healthy nutrition, smoking cessation and mental health protection - stress avoidance, emotional intellectual skill promotion, development and intellectual activity maintenance. Apart from those, other important activities for older persons include: the prevention of sexually transmitted diseases, health instructions by the education system, health in workplace protection as well as occupational diseases prevention, health care within health care and social care - units (healthy senior homes/nursing homes), prevention of infections connected to living conditions in public institutions, the presence of healthy life understood as a value in every community, community relationship support and social integration, information on different health risks and health oriented behaviours, explanation of understanding health determinants and healthy life style in media promotion.

**Table 2:** Roles performed by institutions for HP4OP

SPOFER role	Description of functions performed by institution for a HP4OP programme:
(S) Setting	The given institution constitutes a health promotion setting.
(P) Promoter	The institution (its personnel) implements the programme as street-level promoters, educators, informers or advocates.
(O) Organiser	The institution is responsible for organisation of a given intervention by initiating, providing administrative support, coordinating actions, managing, etc.
(F) Financing	The institution provides funding (entirely or partly) for the given intervention.
(E) Expertise & evaluation	The institution guarantees the proper evidence-based quality of health promotion intervention by providing: guidelines, knowledge, advisement, training, collecting and sharing experiences, but also by evaluating results, etc.
(R) Regulation, monitoring & control	The institution provides legal regulations, monitoring and control: through supervision, registration or by issuing obligatory approval.

**Table 3:** Who undertakes activities in the sphere of health promotion for older people? The institutional analysis scope and sketched results

*Sector*	*MAIN Institutions with health promotion functions indicated for the project purposes*	*Street level health promoters- professionals, health care and public health specialists*	*Place of setting*	*Target groups*	*Particular actions (examples) – where, how*
Health (health care sector understood as involved mainly in diagnostics, treatment, prophylactic processes)	GP/Primary care, Organisations, Insurers	GPs, Nurses, Public health professionals, Physiotherapists	Health centres/units Patient’s homes	Older Patients	Within service delivery – oriented on health conservation, improvement, postponing of worsening health condition, promotion of expected life style (improving health – diet/physical activity recommendation)
		Occupational therapists, Dieticians, Exercise counsellors			
		Pharmacists, Opticians/optometrists, Speech and language therapists			
Education/	Education offices/institutions	Teachers, pedagogy specialists, Sport trainers	Schools, other educational institutions, Sport clubs, Sport centres	Population by age	Educational programme realisation, sport/physical activity support and organisation
Sport					
	Sports organisations/clubs/associations				
Social Assistance	Social Services	Social workers, therapists, officials	Different settings (depending on the particular activity)	Vulnerable older people	Accompanying social service delivery, direct contact with professionals (advocacy for life style/habit change, personal support)
		Environmental nurses			
Governmental	National public health agencies/organs/bodies	Public health professionals, Epidemiologists	Different settings (depending on the particular activity)	Population	Programmes, research, policy/strategy
Regional/Local Authorities	Regional/local public health departments	Public health professionals, Teachers, Play workers, Community workers, Social workers, Environmental health officers	Different settings (depending on the particular activity)	Population by age	Strategies and policies at the local level, activities undertaken by particular professionals at the local level (local government. initiatives)
Enterprise	Health and safety at workplace services (inspectorates), Trade unions and workers organisations, Employers organisations	Occupational medicine specialists	Companies/workplace	Older employees	Regular worker check-ups, diagnostics and other services performed by occupational medical services and professionals, Programmes/trainings organised at the workplace
		Inspectors	Occupational medicine units		
		Activists/educators			
NGO/Voluntary	Social and civic organisations – NGOs	NGO activists, Public health professionals, Trade union safety representatives, Pressure groups	Different settings (depending on the particular NGO and particular activity)	Groups of the older population	Actions of different kinds addressed to the older population in need in different settings (determined by the NGO type and mission)
Media	Media organisations	Journalists - Health correspondents	Different media (press, audio and TV programmes, internet)	The population generally and seniors particularly	Media designing/participating/supporting programmes/actions supporting health promotion for older persons.

(Source: own elaboration)

The institutional and organisational aspect of health promotion is an important part of the wider perspective of public health functions. It is essential in functions such as: the creation of standards, evidence collecting, evaluation and monitoring, financing, communication and cooperation, as well as leadership [4, 30–32]. Definitions adequate to the goal of the study are elaborated for “health promotion” and “intervention”, as well as “institution” itself. This in turn, leads to the development of the analytical framework for the institutional approach to HP4OP. The definitions of health promotion include normative aspects present in international declarations and policy documents but also various (traditional or modern) approaches to health promotion [31, 33]. These definitions have evolved and transformed over decades to accommodate new health challenges [34]. The following proposition was developed within a definitional framework suggested by Jill Maben and Clark [35] as a concise presentation of relevant aspects. It was rearranged and amended with reference to newer publications and international policy documents.

According to the literature review, *health promotion* – as a core function of public health – is the process of improving people’s health status by enabling them individually, and also within a community and through the political system [36] – to increase control over their health and its determinants [37]. Health promotion is a unifying concept – an umbrella term [38] – encompassing various activities (prevention, education, and advocacy) that should: respect human autonomy [31, 33], be sustainable, evidence-based, and be adjusted to the specificity of the target group and local context [36]. It should be performed as a concerted action by various entities from all sectors and on all levels of governance, thus effectively engaging all available resources [36, 39]. A successful health promotion intervention therefore requires the following key functions that are provided by institutions: setting and promoting (core functions) as well as organising, funding, providing expertise and evaluation, and regulation (see Table 2). This definition is devised to encompass the widest possible range of activities actually performed under the term “health promotion” (descriptive aspect), and also recommended activities (normative aspect) in order to be operationalised and serve as an analytical tool – hence the “umbrella approach” [38]. It focuses not on activities but rather on issues of management, organisation, actors’ involvement, functioning, etc. Thus, there is a strong incorporation of policy-oriented definitions [36, 39] that contain a set of key organisational functions or roles that institutions can perform in HP4OP.

As seen in the literature review, health promotion *activities* (the processes of health promotion as defined above) can take various forms. The most general is *intervention*, i.e., “interference” in the current state of affairs – an intrusion aimed at change (behavioural, social, environmental or policy change). In this case, health promotion is not clearly distinct from other activities. However, the most organised and clearly distinguishable form is the health promotion *programme* – a set of organised activities strictly designed with the health promotion objective in mind, which are limited in terms of their scope, organisation, engaged actors and duration.

For the purpose of this study, the choice was made to define an *institution* as “an interlocking double-structure of persons-as-role-holders or office-bearers and the like, and of social practices involving both expressive and practical aims and outcomes” [2]. It should be noted that in the health promotion literature, the term “institution” is usually restricted to organisations providing social or other types of care on a daily basis, including those that are based on involuntary commitment, like psychiatric wards or prisons: “penitentiary institutions” [40]. The latter are considered as one of the possible settings for health promotion. Furthermore, the term “institution” occurs in concepts like “institutional setting” as opposed to workplace setting, community setting or health facility setting [41]. A definition of an institution as an organisation [42] need to be adopted in the analysis of HP4OP for the identification of institutions that perform crucial roles towards the realisation of health promotion activities for older people. The focus should be on formal organisations – those with defined objectives; but without the exclusion of the informal ones, since they can also be involved, even in formalised programmes. Not only are homes and local communities frequently a setting for health promotion, but also informal networks and groups that are involved in organisation and promotion. Overall, health promotion is a core part of the work of many different institutions, inside and outside the health sector, which operate in different political, economic, social and legal circumstances (see Table 3).

### Literature review results: systemic context

An important result of the literature review was the overview of the political and historical context of HP4OP in Europe. The traditions of different socio-economic approaches (models of welfare-states) and institutional structures of political systems shape the level of commitment to the most extensive approaches to health promotion (health advocacy). These factors determine which institutions (belonging to which sectors and levels of governance) are involved in health promotion. In this context, scholars classify welfare states as “social democratic” (Nordic countries), “liberal” (e.g., UK), “Latin” (e.g., Italy, and Greece), and “conservative” (e.g. France, Germany, and Belgium), which is based on the modified Esping-Andersen classic typology of social policy models [43, 44]. However, post-Soviet countries also have their own set of experiences rooted in the Semashko model of health-care system [45, 46].

In some countries, the main responsibility for health promotion is attached to the central government; in others to the territorial self-government. The involvement of various sectors depends on the approach. For instance, “conservative” and “liberal” states often utilise the participation of non-governmental and even for-profit private institutions whereas “social-democratic” Scandinavian countries prefer the involvement of decentralised public authorities [43]. Also, in Central and Eastern Europe and the countries of the former Soviet Bloc, health promotion was, and still is, neglected [45]. This remains a problem even despite a strong focus on preventive medicine (secondary prevention, often ineffective screening [46]) and the significance of the SANEPID health promotion services (primary prevention) [47, 48]. It is perhaps due to the association of “public health” with the central public authorities that creates barriers to inter-sectorial action. Significant disparities can be observed here, not only in respect to economic development but also regarding political ideologies that have been determining the health promotion strategies for decades. This impact has lasted long after the systemic transitions [49]. Central and Eastern European countries – when compared to the “old” EU countries – have underdeveloped and fragmented health promotion. Consequently, little attention is paid to non-communicable diseases and mental health [47]. The problem of disparities concerns not only activities but also appropriate expertise and research on health promotion [49].

The literature review also suggests that currently, governing political parties shape the approach to health promotion. Economically liberal and conservative governments tend to appreciate individualistic approaches that focus on health education and individual lifestyle choices while opposing health advocacy that could actually make healthy choices easier. This policy orientation drives those countries to a stronger emphasis on the role of NGO and commercial sectors but on contractual and non-obligatory terms. Such a tendency is more common than international declarations would suggest [50]. While declaratory commitment to health promotion is strongest in social-democratic (Nordic countries) and “liberal” (e.g. UK) states, the latter are less likely to put policies into practice [49]. Identification of health promotion leadership at a national level is problematic due to significant local differences [51].

An important issue related to the legal basis in respect to the research undertaken, is the creation of a health promotion glossary defining crucial terms and frames in this area [52]. Such glossary is necessary to provide terminology that should be used in legal documents and can have a supranational harmonising impact, stimulating the new approach to public health [53]. In terms of legislative actions, laws on health, public health and health promotion originally served to define and justify public health measures and the scope of the responsibility of the state in this area. In this context and regarding the supra-national level, very general international acts have to be mentioned with particular attention to the WHO international legal initiatives: The Ottawa Charter and The Bangkok Charter [36, 39], as well as the 2009 WHO’s *Milestones in health promotion* [54].

In the 21^st^ century, another influential element of legislation can be identified, namely the cross-sectorial impact on health, in accordance with the “Health in All Policies” idea, based on the World Health Declaration. Recently, in 2015 at the 67th World Health Assembly (Resolution WHA 67.12), WHO requested the WHO Director-General to prepare, for the consideration of the 68th World Health Assembly, a “Framework for Country Action Across Sectors for Health and Health Equity”, which could be used for different purposes, with regard to the Health in All Policies document. Accordingly, a draft framework was developed in three rounds of informal consultations, which is expected to culminate successfully in a form of a new international regulation [55].

Regarding internal legislation however, only a minority of European countries (mainly in northern Europe) have established laws that relate specifically to health promotion. As an example, the Finnish legislation should be highlighted [56]. This act emphasises, among other things, that the “*Health During Working Life* and *Health in Old Age policies, including the psychophysical demands of work, are health-promoting and appropriate for workers of different ages and preconditions for promoting the health of older people and for reducing the health differences created by reducing prejudices and attitudes contributing to age discrimination”*. With regard to older workers in particular, the fact that they have increased in number and gained importance only in recent years, explains that existing promotion programmes specifically targeted at this group of people are not sufficient [57].

### Literature review results: overview of activities and institutions

Following the literature review on country specificity, the emerging picture of the selected countries’ institutional structures for HP4OP is quite incomplete and heterogeneous. The scientific literature review showed that a disproportionally large amount of institutional information is available on the Netherlands and Germany. At the same time very little is provided on Hungary, Portugal, Bulgaria and Lithuania. Often, most notably in the scientific literature, a precise identification of the number of institutions involved was not possible due to the vagueness of information. In some cases, only a group of institutions in the same category was recorded. Also, a lack of sources concerning the issue in some countries does not always signify a lack of such activities. This also results from a shortage of substantial evaluation of HP4OP in given countries, or a lack of publicly available report on such evaluations (at least in English).

The literature review indicates an abundance of literature devoted to the characteristics, efficiency and performance of various HP4OP programmes. Scientific papers usually focus on the content of health promotion rather than on its institutional arrangements. Interestingly, the grey literature and project web databases provided much more substantial information on the issue, whereas scientific literature usually delivered a mostly sketchy and incomplete picture of institutional arrangements since it was not dedicated to institutional analysis. Eventually, the literature review shows that the grey literature and dedicated health promotion databases, as well as ongoing pilot surveys and interviews, are much more effective data-sources on institutions involved in health promotion for the older people in the analysed countries.

As expected, the literature review points out seven main sectors potentially involved in health promotion, and specifically health promotion addressed to the population aged 65+ (except for the workplace sector): (1) health sector, (2) social sector (3) central and local government, (4) workplace/enterprises/employers, (5) NGOs & voluntary organisations, (6) sport & education, and (7) media. A great degree of inter-sectorial cooperation is identified. Frequently programmes engage more than one institution and often, these institutions belong to different sectors.

### Questionnaire survey results

Experts’ opinions – supplemented by the literature review results – helped to identify the leading sectors, i.e. those sectors that are (practically and formally) more engaged in or responsible for HP4OP than others in each of the analysed countries. This reveals quite a complex picture of the diversity of sectors and organisations/institutions active in this field in the countries.

The results of the questionnaire survey reveal a few quite interesting details. First, the presumption of a diversity of sectors and categories of organisations most engaged in HP4OP in the analysed countries is confirmed. Next, the sectors that are most frequently indicated as being principally active in HP4OP in the majority of cases are:

- Regional/local authorities (in 9 countries): Germany, Italy, the Netherlands, Poland, Bulgaria, Greece, Hungary, Portugal and Lithuania
- Health sector (in 8 countries): Germany, Italy, the Netherlands, Poland, Bulgaria, Greece, Portugal and Lithuania.
- Voluntary/NGO organizations (in 5 countries): Poland, the Czech Republic, Greece, Portugal and Hungary.

A general overview of those results are presented in Table 4. This table shows a summary of results: the most active sectors in each country are marked as "Most important" while sectors in given countries that are still active in HP4OP, but of less importance, are marked as "Important". As shown in the table, the sectors indicated as less relevant are: enterprise (2 countries: Italy and the Netherlands) media (3 countries: the Netherlands, Bulgaria and Lithuania) and education & sport (5 countries: Germany, Italy, the Netherlands, the Czech Republic and Lithuania). In all these cases, there are only weaker oriented (secondary) HP4OP activity compared to those indicated as most relevant. Interestingly, the social assistance sector is only indicated once as the most active in HP4OP (in the Czech Republic), plus 4 times in a secondary position (in the Netherlands, Bulgaria, Greece and Lithuania).

**Table 4:** Outline of the most important sectors for HP4OP in selected EU countries

		1.	2.	3.	4.	5.	6.	7.	8.	
	SECTOR→	Health	Education & Sport	Social Assistance	Government	Regional / Local	Enterprise	Voluntary / NGO	Media	*Sectors chosen for further analysis - per country* *(number of sectors chosen)*
	Country:									↓
*I*	BG	Most important		Important		Most important		Important	Important	Health, Government, Regional/Local (3)
*II*	CZ	Important	Important	Most important	Important	Important		Most important		Social Assistance, Government, Voluntary/NGO (3)
*III*	D	Most important	Important		Important	Most important				Health, Education & Sport, Government (3)
*IV*	GR	Most important		Important	Important	Most important		Most important		Health, Government, Voluntary/NGO (3)
*V*	H				Important	Most important		Most important		Government, Regional/Local, Voluntary/NGO (3)
*VI*	I	Most important	Important		Important	Most important	Important	Important		Health, Government, Regional/Local (3)
*VII*	LT	Most important	Important	Important		Most important		Important	Important	Health, Government, Voluntary/NGO (3)
*VIII*	NL	Most important	Important	Important	Most important	Most important	Important	Important	Important	Health, Education & Sport, Government (3)
*IX*	P	Most important			Most important	Most important		Most important		Health, Government, Regional/Local (3)
*X*	PL	Most important			Important	Most important		Most important		Health, Government, Regional/Local, Voluntary/NGO (4)
	*Number of counties where a given sector has been chosen for analysis*	8	2	1	10	5	-	5	-	

The table also shows the level of diversity of sectors engaged in HP4OP in each of these countries, according to the survey. For example, the Netherlands, the Czech Republic, Lithuania and Italy seem to have these activities spread over a range of different sectors (as many as 6–8), while in other countries they are “concentrated” in fewer sectors, e.g. in Hungary (2 sectors) or Poland (3 sectors). Only in the case of Germany, one additional sector is indicated under the category of “o*thers*”: namely, National Cooperation Networks (National Health Targets, Equity in Health, Healthy Cities Network). No other sector is added to the list in any of the analysed countries, which may indicate that the range of sectors chosen for HP4OP analysis is comprehensive.

There is a range of national regulations concerning HP4OP issues, such as the national strategy of healthy ageing, for example in Italy, Bulgaria and Poland. In other countries, such documents are under construction (Greece). In yet other countries, there are already nationwide long-term national health programmes (strategies, plans), including references to HP4OP, for example in Germany and Lithuania. However, there are a few countries where there are no such dedicated official documents, such as the Czech Republic and Hungary.

Opinions about the implementation of the contents of such documents in practice differ from country to country, but in several of them, for example in Bulgaria, Germany, Greece, Italy and Poland, the effects are reported to be partial and the results limited. A low level of financing, the lack of staff and inefficient coordination is used to explain this. In the Netherlands, the situation seems to be better due to the government commitment and subsidies dedicated to different aspects of HP4OP.

The funding of HP4OP activities is attributed to both central and regional governments, and is supplied through taxation or by national health insurance/funds in the majority of the countries (Bulgaria, Greece, Hungary, Italy, Lithuania, the Netherlands and Poland). NGOs and different foundations are reported as being involved in HP4OP financing as well, for example in Bulgaria, Greece and the Netherlands. In some countries, these organisations were indicated as beneficiaries of public funds and/or EU grants (the Czech Republic, Greece, Lithuania, and the Netherlands). Germany has strictly defined funders of health promotion in general: statutory health insurance, public budgets, statutory accident insurance, employers and private households.

The application of the concept of *evidence-based knowledge* is rather rarely reported regarding the evaluation of health promotion in general and HP4OP programmes in the investigated countries. Only in a few cases, there are dedicated national organisations (agencies) responsible for setting standards and assessing such programmes (in Germany, Lithuania and, to some extent, in the Netherlands).

This survey – especially its final question – indicated several additional sources of information on the survey topic. The expert respondents however point out that although many organisations even issue their internal evaluation documents in English, a number of these sources, especially on the local level, are mostly in the original, native languages and are not translated.

### Combined results on organizations involved in HP4OP and their analysis (review and survey)

The survey results are consistent with the presumption that the European countries approach HP4OP differently. Also the involvement of different sectors and a broad range of institutions and providers has been confirmed. Such a multi-faceted picture corresponds to the countries’ diversity in the organisation of health systems as well as other national systems (regulatory, administrative, economic, social, etc.). HP4OP is also executed at diverse levels of government and administration (central, regional and local) with a varying degree of involvement [1, 3, 11, 16, 17, 23–29, 58, 59].

It should be noted that the questionnaire results described above do not have stand-alone value. They may be treated as an important, but only partial contribution. Together with the literature review, the results create the preliminary picture of the institutional approach to HP4OP in the analysecd countries. It seems that such a combined approach advances knowledge and can set the direction for further, in-depth research on this vital issue of health promotion for older people in European countries.

Thus, based on the literature review and questionnaire survey results, a set of organisations, most involved in HP4OP have been identified in the countries of interest. This comprises the following sectors per country:

- Bulgaria-Health, Government, Regional/Local Authorities
- Czech Rep.-Social Assistance, Government, Voluntary/NGO
- Germany-Health, Education and Sport, Government
- Greece-Health, Government, Voluntary/NGO
- Hungary-Government, Regional/Local authorities, Voluntary/NGO
- Italy-Health, Government, Regional/Local Authorities
- Lithuania-Health, Government, Voluntary/NGO
- Netherlands-Health, Education and Sport, Government
- Portugal-Health, Government, Regional/Local Authorities,
- Poland-Health, Government, Regional/Local Authorities, Voluntary/NGO

It should be mentioned however that the sectors listed for each country do not fully overlap with the survey results. This is an aggregate result of not only a confrontation with the literature review, but also a consequence of certain methodological reasons. Above all, the decision was made to include the analysis of the central government sector for all countries. In the case of Germany and the Netherlands, the education and sports sectors replaces the local self-government sector. In case of Lithuania the NGO’s sector is selected as relevant as well. Media and enterprise sector are not defined as the most important factor in HP4OP in any country.

The presented institutional approach provides guidance for further analyses of the HP4OP institutions with the sectors listed above. Within the health promotion definition (see above), a set of key institutional functions – required for successful HP4OP intervention – can be identified: setting (S), promoting (P) (core functions) as well as organising (O), funding (F), providing expertise (E) and regulating (R) with evaluation and control (see Table 2). These sets of functions together form a framework (SPOFER) that can serve future studies in two ways: firstly as a classification of roles that institutions can perform for any given HP4OP programme and – secondly – as a checklist of key roles required for any HP4OP programmes.

## Discussion

The analysis presented here, concerning the institutions providing HP4OP in the selected European countries, undertaken with the use of a literature review and survey among experts, provides results relevant to both future research and policy. We find that the scientific literature provides a sketchy and incomplete picture of HP4OP. In contrast, the grey literature is a much more substantial source on the institutional dimension of HP4OP. We also find that there are significant HP4OP organisational differences in the group of analysed countries, linked to systemic differences: political, welfare state models, etc. Inter-sectorial cooperation is common in all countries as also confirmed in the survey. The need of a cross-sectorial approach is thus recognized.

The original institutional approach applied in the present study has provided a clearer and more systematised picture of health promotion activities in terms of the institutions involved and roles they play. The literature review performed together with the survey among the group of experts from the ten European countries has provided a still limited, but already indicative picture of the sectors and institutions involved in HP4OP in these countries. Seven main sectors are potentially involved in health promotion: (1) health sector, (2) social sector (3) central and local government, (4) workplace/enterprises/employers, (5) NGOs & voluntary organisations, (6) sport & education, (7) media. The most engaged sectors in HP4OP in the examined countries have proven to be: health care, regional/local authorities and NGO’s.

The importance of this study concerns not only a wide range of results from the studied countries but also a comparative perspective on the institutional arrangement of HP4OP in Europe. This, at least partially, fills an existing gap in respective knowledge as outlined at the outset of the paper. The results confirm the main assumptions for a cross-sectorial approach to public health and HP4OP. Moreover, the role of different institutions and inter-institutional arrangements in HP4OP was stressed as fundamental in the study results. The final selection of sectors per country outlined at the end of the results section can be used for further in-depth analysis to better understand the health promotion activities in the selected countries.

In view of the increasing trend of aging workforces, it is necessary to update information about policies and programmes implemented in the field of workplace health promotion in European countries. Taking into account the influence of the workplace during the final years of professional activity, and the obviously growing role of the media in the process of providing information and promotion of a healthy lifestyle, it is important not to leave out these two sectors in further research. Also, the central government (represented usually by the Ministry of Health, the Ministry of Labour and Social Policy, the Ministry of Sport, and the National Institutes of Public Health [or the given countries’ equivalents]) is important to be studied in each of the countries. The reason is the key role in public health that is usually attributed to this stakeholder as well as the expectation to obtain overarching, complementary guidelines for in-depth country specific research. Consequently, the next relevant stage of research is considered to be a comprehensive analysis of the most important sectors that are active in HP4OP in each of the countries, as listed above. Also, it is assumed that central governments – as supervisory and regulatory institutions – can provide a representative overview of the situation in each country. Through those institutions, we expect a further snowball effect in data collection.

The engagement of experts from the investigated countries turned out to be exceptionally helpful as a complementary source of knowledge to the results of the literature review. Their opinions revealed aspects which enable properly fine-tuning actions in the next steps of research. Moreover, elaboration of a relatively complete and reliable picture of HP4OP in a given country without the support of local expert knowledge concerning the specificity of the country’s situation and solutions, would not have been possible. The limits concerning research results, however, still have to be accounted for, namely the native language of some sources, the absence of data concerning specifically HP4OP in the literature and the overall problem of the non-existence of a common model for HP4OP activities.

Certain study limitations grow out of the specificity and interdisciplinary characters of the issue. Most of those were indicated in the results and method sections. The authors believe that the evidence presented here forms quite a solid foundation for further in-depth analyses, which are planned as the next steps of the Pro-Health 65+ project.

## Conclusions

The institutional approach applied in the present study has provided a clear and systematised picture of health promotion activities and institutions, specifically in the HP4OP sphere. This approach to HP4OP also enables the identification of existing knowledge gaps in this area that can be addressed in further research. The results show the necessity of adopting a cross-sectorial approach to explore the role of different institutions and inter-institutional arrangements in HP4OP. The engagement of country experts to supplement the literature review concerning the HP4OP in individual countries proved to be a well-reasoned approach. It can provide guidance and points of reference for furture in-depth analysis.

According to this study, the sectors most engaged in HP4OP are: health care, regional/local authorities and NGO’s/voluntary organizations, all of them also being strongly related to each other regarding health promotion activities. Health programmes are often implemented with the participation of all three sectors as they are responsible for different aspects and cooperate in respect to similar or common tasks. Interestingly, the role of the social assistance sector has been emphasised in a few of the analysed countries, as being most important for HP4OP, and in a majority of cases, as being of secondary importance.

According to the results of this study, the workplace is not considered the most relevant institution in HP4OP interventions. Nevertheless, the scientific and grey literature review indicates the essential problem: at the workplace, age discrimination should be eliminated through adequate policies, the promotion of lifelong learning, the adaptation of work demands and the environment and the promotion of health and wellbeing. These should serve to ensure a longer working life and higher employment rates amongst older workers.

Furthermore, it is difficult to unequivocally determine the role of the media in HP4OP. The literature review clarified the issue to some extent, namely the aggregated scientific evidence showing global or focused media interventions in the analysed countries does not exist. Neither did experts indicate media as an important factor for HP4OP in any of the analysed countries. Bearing in mind the specificity of HP4OP and the group of beneficiaries as main addressees of TV and radio programmes, further studies are necessary in this context. A dedicated research tool and the support of local experts would be necessary, as well as deeper desk research.

There are not many examples of separate HP4OP actions within the sport sector, although a rising awareness of the importance of this area may be observed. Still, a few very good examples of sport projects dedicated to supporting and enhancing older people’s health in some countries may be indicated. At the stage of research reported here, it is assumed that HP4OP is rather “spread out” amongst sport and educational health promotion activities that are undertaken by both governmental and non-governmental bodies. Again, this point is to be more deeply examined in future studies.

## Abbreviations

GP, general practitioner, family doctor; HP4OP, health promotion for older people; NGO, non-governmental organisation; SANEPID, sanitary & Epidemiological surveillance (a body originating from the health systems of Soviet Bloc countries).
